# Reliability and External Validity of AMSTAR in Assessing Quality of TCM Systematic Reviews

**DOI:** 10.1155/2012/732195

**Published:** 2012-02-16

**Authors:** Deying Kang, Yuxia Wu, Dan Hu, Qi Hong, Jialiang Wang, Xin Zhang

**Affiliations:** ^1^Department of Evidence-Based Medicine and Clinical Epidemiology, West China Hospital, Sichuan University, Chengdu 610041, China; ^2^Department of Medical Information, Zigong Third People's Hospital of Zigong City, 643000 Zigong, China

## Abstract

*Objective*. The aim of this study is to measure the reliability and external validity of AMSTAR by applying it to a sample of TCM systematic reviews. *Study Design and Methods*. We tested the agreement, reliability, construct validity, and feasibility of AMSTAR through comparisons with OQAQ. Statistical analyses were performed by using SPSS 13.0. *Results*. A random of sample with 41 TCM systematic reviews was selected from a database. The interrater agreement of the individual items of AMSTAR was moderate with a mean kappa of 0.50 (95% CI: 0.26, 0.73). The ICC for AMSTAR against OQAQ (total score of 9 items, excluding item 10) was 0.87 (95% CI: 0.76, 0.93). *Conclusions*. Although there is room for improvement on few items, the new tool is reliable, valid, and easy to use for methodological quality assessment of systematic reviews on TCM.

## 1. Introduction

Traditional Chinese medicine (TCM) is one of the rarely existing traditional medicines that hold systematic theories as well as preventative and therapeutic methods for diseases in practice [1]. Since the 1950s, research methods in modern medicine (Western medicine) have been gradually introduced to TCM studies; the first TCM systematic review appeared in medical journals in the late 1990s, and now hundreds to thousands of reviews have been published on the area of TCM [2, 3]; systematic reviews have become the standard approach in assessing and summarizing primary studies. However, even though systematic reviews proved to be useful, serious consideration must be given to how they were conducted; high methodological quality of systematic reviews is a prerequisite for recommendation of the use or avoidance of a TCM intervention. At present, either researches or methods for assessing quality of systematic reviews are still not fully developed in TCM and there is substantial room for improvement [2, 3]; even in Western medicine current instruments for assessing methodological quality of systematic reviews are still suboptimal and need revision and updating [4, 5]. A new tool termed AMSTAR, an acronym for “a measurement tool to assess systematic reviews,” was developed strictly upon the OQAQ (Overview Quality Assessment Questionnaire) [6], the Sack checklist (quality assessment checklist) [7], and three additional items. This tool is an 11-item questionnaire requiring assessors to answer “yes,” “no,” “cannot answer,” or “not applicable”; and a recent study reported that AMSTAR has good agreement, reliability, construct validity, and feasibility to assess the quality of systematic reviews [8]. However, these psychometric properties within AMSTAR were tested by having it apply to only a limited set of systematic reviews from Western medicine; a further step is needed to assess its validity and reliability, by a broader range of assessors and more samples of reviews in diverse circumstances [8]. Will the results be reproducible when assessing methodological quality of systematic reviews on traditional chinese medicine (TCM)? The answer is not clear yet, further validation is necessary. The aim of this study is to validate the reliability and external validity of AMSTAR by applying it to a sample of TCM systematic reviews.

## 2. Materials and Methods

### 2.1. Identification and Inclusion of Systematic Reviews on TCM

We have adopted the definitions used by the Cochrane Collaboration: a systematic review is a review of a clearly formulated question, that which uses systematic and explicit methods to identify, select, and critically appraise relevant researches, and to collect and analyze data from the studies included in the review [9]. Then a search strategy for locating systematic reviews on TCM was formulated by using the definitions; search terms included “Chinese medicine”, “Chinese herb”, “plant preparations”, “Chinese medical formula,” “meta analysis,” “meta-analysis,” “meta-analyses,” and “systematic review.” We performed a comprehensive search on CNKI (China National Knowledge Infrastructure), CBM (Chinese Biomedical Database), VIP (Chongqing VIP periodicals Database), Medline and EMbase databases (January 1, 1999 to the end of 2008) in addition to hand search on Chinese Journal of Evidence-Based Medicine (incept to the end issue of 2008), Chinese Journal of Integrated Traditional and Western Medicine (from the first issue in 1999 to the end issue in 2008), and Journal of Chinese Integrative Medicine (from the first issue in 1999 to the end issue in 2008). Two reviewers screened the titles and abstracts of identified studies independently, disagreement was resolved by discussion. A total of 165 systematic reviews on TCM were included, full detail of evaluations has been reported separately [10]. We used a computer-generated random sample (approximately 25% of the 165 systematic reviews) as a test set for validation.

### 2.2. Data Extraction

We tested the agreement, reliability, construct validity, and feasibility of AMSTAR through comparisons with OQAQ, the latter was a validated scale (overview quality assessment questionnaire, OQAQ) developed by Oxman and Guyatt in 1991 [6]. The OQAQ scale measures across a continuum using nine questions (items 1–9) designed to assess various aspects of the methodological quality of systematic reviews and the tenth item requires assessors to assign an overall quality score on a seven-point scale [6].

In order to adhere more faithfully to the guidance provided by AMSTAR and OQAQ, two assessors (KDY, WY) performed a separate translation and conducted a pilot test independently. Each translator prepared a separate translation and the difficulty in obtaining conceptually equivalent expressions in Chinese was assessed too; subsequently, a sample of 2 systematic reviews on TCM was taken to perform pilot test by two authors independently; based on the results and consensus from reviewers, the final Chinese versions of AMSTAR and OQAQ were then developed for formally evaluating methodological quality of Chinese systematic reviews; all inconsistencies identified either in translation or in application were resolved by discussion. On the basis of this, we constructed a data extraction form in Chinese for this study, in which 11 items of AMSATR and 10 items of OQAQ were adopted directly. In addition, publication status and reporting characteristics of systematic reviews, such as publish language, number of pages, funding source, update or not, Cochrane systematic review or not, target disease, and institution of first author, were also incorporated; besides, the time required to complete an assessment while applying AMSTAR and OQAQ was recorded too.

### 2.3. Agreement and Reliability

Assessors were required to answer “yes” score or any other scores for each of items (AMSTAR and OQAQ). If an item was scored “yes,” it would be given one point, otherwise, 0 point. We added up these to calculate a total score, the reliability of this total score was assessed through calculating intraclass correlation coefficients; the agreement for each item and the overall tool was explained by percentage of actual agreement as well as Kappa coefficient [5, 8]. Kappa coefficient is a popular measure for chance-corrected nominal scale agreement between two raters. We adopted the Kappa values of <0 rates as less than chance agreement, 0.01–0.20 as slight agreement, 0.21–0.40 as fair agreement, 0.41–0.60 as moderate agreement, 0.61–0.80 as substantial agreement, and 0.81–0.99 as almost perfect agreement [8].

### 2.4. Validity and Feasibility

The OQAQ was selected as a criterion tool because it had been rigorously developed, its face validity was strong, and its validity had been thoroughly tested [6]. We assessed construct validity by comparing AMSTAR, OQAQ, and a self-developed global assessment scale. Construct validity was showed by intraclass correlation coefficient (ICC); for the purpose of calculating ICC, we adopted methods used by the AMSTAR group [8], and converted the mean total scores (mean of two assessors) of per review to the percentage of maximum score (11 points in AMSTAR and 9 points in OQAQ accordingly); in addition, we developed a 100-point rating scale for overall quality assessment based on answers to the eleven questions in AMSTAR, in which two assessors indicated his or her judgments by checking tick-marks on a horizontal line (0 to 100 point), an SR without any flaws would be scored 100 points. Meanwhile, we also adopted the item 10 in OQAQ as a validated global assessment instrument. The overall mean scores (mean of two assessors), either using the self-developed 100-point rating scale or using the item 10 from OQAQ, were also taken to verify the construct validity of AMSTAR. The feasibility of AMSTAR was assessed by recording the time it took to complete scoring, and paired *t*-test or nonparametric test was applied when comparing with OQAQ.

Database was established by using an electronic form on Microsoft Excel 2003 (Microsoft Corp., Redmond, WA); the data set extracted contained two quality ratings for each review, yielding a total of four ratings per review. Data analysis was performed by SPSS 13.0 (SPSS, Chicago, IL). *P* < 0.05 was considered significant.

## 3. Results

A random sample with 41 TCM systematic reviews was selected from a database developed in a previous study [10]. Of which, only 9 reviews were written in English, and the majority (78%) was published in Chinese journals [11–51]. The sample included 35 paper-based reviews and 6 Cochrane reviews; there was only one updated Cochrane review. According to the International Classification of Diseases 10 (ICD-10), the topics of the reviews ranged across 9 systems, and mainly focused on diseases of circulatory system (15 SRs or 37% of the sample, such as stroke and other cardiovascular diseases), infectious and parasitic diseases (7 SRs, like HBV, SARS), genitourinary system (5 SRs, such as ectopic pregnancy), digestive system (4 SRs, like ulcerative colitis), nervous system (3 SRs, such as Parkinson's disease), and musculoskeletal system and connective tissue (3 SRs, osteoporosis). The number of pages of included TCM SRs ranged widely from 2 to 80 with a median of 6 pages, of which, Cochrane systematic reviews had more pages than non-Cochrane reviews (*P* < 0.001), with medians of 31 (range: 16–80) and of 5 (range: 2–11), respectively. Less than half of the reviews (41.5%) were presented by clinicians.

Total mean scores on AMSTAR ranged from 2 to 10 (out of a maximum score of 11) with a mean percentage score of 55.1%. The total mean quality scores on OQAQ ranged from 3 to 8 (out of a maximum score of 9) with a mean percentage score of 63.6%. The overall scores for the global assessment instrument (item 10 in OQAQ) ranged from 1 to 6 (out of a maximum score of seven) with a mean of 3.3 (95% CI: 2.9, 3.6), and overall scores using the self-developed 100-point rating scale ranged from 15 to 73 with a mean of 47.6 (95% CI: 43.4, 51.7).

### 3.1. Agreement and Reliability

Substantial agreements (>80% or nearly 80%) were observed in majority of the individual items (item 1, 2, 3, 4, 6, 7, 8, 10, 11) on AMSTAR, with a mean percentage of 84% (95% CI: 71.1%–91.9%); agreements on item 5 and item 9 were mild at a percentage of less than 60%. Kappa ranged widely from −0.03 to 1.00 with a mean of 0.50 (95% CI: 0.26, 0.73); five items (item 2, 3, 4, 10, 11) scored a kappa of >0.70 (0.70 to 1.00); the highly agreements in four items (item 1, item 6, item 7, item 8) ranged from 80% to 95% and inversely low kappa (−0.034, 0.40, 0.36, 0.36) may be explained by a skewed distribution of responses, that is, approximately 80% (in item 6) to 90% (in item 1, 7, 8) of reviews in which the assessors agreed on the score “yes.” However, items 5 (list of included and excluded studies) and 9 (appropriate method to combine studies) scored relative low either in agreement percentage or in kappa parameter (Table 1). Agreements on individual items of OQAQ were inferior to AMSTAR, with a mean percentage of 79% (95% CI: 64%, 89.5%), and the mean of kappa was relatively low too, with a mean of 0.35 (95% CI: 0.08, 0.62).

The interobserver ICC to the total score was excellent for AMSTAR at 0.84 (95% CI: 0.70, 0.91), and superior to OQAQ, 0.67 (95% CI: 0.38, 0.82). The interrater agreement (kappa) between two assessors for the global assessment (item 10 in OQAQ) was 0.72 (95% CI: 0.48 to 0.85), and better than the self-developed 100-point rating scale: 0.50 (95% CI: 0.07–0.74).

### 3.2. Construct Validity

Total mean score was converted into the percentage of the maximum score for each of the instruments, the ICC for AMSTAR against OQAQ (total score of 9 items, excluding item 10) was 0.87 (95% CI: 0.76, 0.93), that is, the results of AMSTAR were highly convergence with the results of OQAQ. Besides, both overall scores were converted into the percentage of maximum score. ICC obtained when comparing AMSTAR with the item 10 in OQAQ was 0.84 (95% CI: 0.69, 0.91), and when comparing with the 100-point rating scale, ICC was at 0.81 (95% CI: 0.65 to 0.90) respectively; thus AMSTAR showed well convergence with global assessment instruments too.

In addition, we compared the total scores obtained by applying AMSTAR on Cochrane reviews (*n* = 6, 8.42 ± 1.02) with that of non-Cochrane reviews (*n* = 35, 5.66 ± 1.31), and the former had higher quality score than the latter (mean difference = 2.76, *P* < 0.001, 95% CI: 1.62, 3.90). The relationship between quality scores and publish year was explored too, reviews published after 2005 had similar AMSTAR scores comparing to earlier reviews (5.98 ± 1.51 versus 6.18 ± 1.76, *P* = 0.70). As the methodological quality and the reporting quality were not mutually exclusive addressing in AMSTAR [8], we explored whether the number of pages had a positive or negative effect on the AMSTAR score, the result showed there was a statistical association between AMSTAR score and the number of pages (Spearman's rho = 0.67, *P* < 0.001).

### 3.3. Feasibility

It took 13.2 (95% CI: 12.2, 14.2) minutes to complete use of AMSTAR for each review, while it took less time to complete scoring of OQAQ, averagely 9.3 (95% CI: 8.8, 9.9) minutes per review (paired difference = 3.9, *P* < 0.001). Besides, a linear regression analysis was performed (time = 6.35 + 2.94 × langrage + 1.75 × log⁡⁡(pages), *P* < 0.001), revealed the time needed to complete using AMSTAR had significant associations with logarithm of the number of pages (unstandardized coefficients = 1.75, 95% CI: 0.61 to 2.90) and langrage (unstandardized coefficients = 2.94, 95% CI: 0.73 to 5.15); that is, systematic reviews with more pages or written in English need more time to complete scoring. The two assessors found there's difficulty in approaching a final decision on item 9 “were the methods used to combine the findings of studies appropriate” and item 5 “was a list of studies (included and excluded) provided”; for the latter, more detailed guidance for scoring “yes” are required.

## 4. Discussion

A considerable amount of systematic reviews on traditional Chinese medicine have been conducted since the first TCM systematic review was published in the late 1990s [2, 3]. We selected a random sample of TCM systematic reviews from a database developed in a previous study [10], the sample covered a wide variety of health topics, and thus, we believe that we had a representative sample of TCM reviews of which the AMSTAR was ready to apply.

Our findings in this research revealed that the AMSTAR is a good choice for evaluating quality of TCM systematic reviews. The AMSTAR showed satisfactory interrater agreement, convergent validity, and feasibility in assessing methodological quality of TCM systematic reviews.

Interrater reliability was evaluated by assessing the degree to which different individuals agreed on the scientific quality of a set of reports [7, 8], the performance of AMSTAR in terms of agreement and reliability was better than that of OQAQ; overall agreement and kappa of items in AMSTAR ranged from moderate to perfect, the reliability of its total score was excellent. However, fair agreement and relatively low kappa were observed in item 9 “were the methods used to combine the findings of studies appropriate” and item 5 “was a list of studies (included and excluded) provided,” indicated that there is a room for improvement on AMSTAR when applying this new tool to assess methodological quality of systematic reviews on TCM.

In the absence of a gold standard, we assessed construct validity by comparing AMSTAR with OQAQ as well as two global assessments. Construct validity was shown by intraclass correlation coefficient (ICC); this statistic reflects the extent to which the results of AMSTAR converge with the results of other “criterion” instruments. The analysis revealed that the construct validity was excellent, that is, the AMSTAR is a reliable and valid tool. Given the extremely strict implementation of Cochrane systematic review, such reviews conducted with high methodological quality have been widely recognized [52]; the AMSTAR revealed Cochrane systematic reviews have higher quality scores than non-Cochrane systematic reviews, that is, the AMSTAR has an ability to discriminate methodological quality, so it is sensitive when applying it to a sample of systematic reviews in diverse quality. The relationship between AMSTAR quality score and the number of pages can be explained by the fact that Cochrane reviews always have considerable amount of pages, these extreme outliers determined the direction and strength of the association.

Compared with the results reported by Shea et al. [8], considerable differences exists either on ICC for AMSTAR contrast to the OQAQ, or on items with low kappa values. Shea et al. reported a lower ICC of 0.66 (95% CI: 0.28, 0.84), and different items (item 4 and item 7) with relatively low kappa parameter. Possible explanations for these differences include (i) the different samples of systematic reviews being evaluated, most SRs in our sample were short, published in Chinese, and more likely to be rated as low quality, such less conversely evaluations could produce a higher ICC value in our study; (ii) the extra procedure of translation and the conducts of evaluations by applying two tools simultaneously may act as a kind of consensus training to make the evaluations of AMSTAR more likely convergent with the results of OQAQ, that lead to a higher ICC value too in the present study; (iii) the background, skills, and expertise of the assessors were different from that of Shea et al. study.

Regarding the feasibility of AMSTAR, the time needed to complete scoring showed this new tool is feasible in assessing quality of TCM systematic reviews too; it took about 13 minutes on average to complete an assessment and showed well applicability. The statistical analysis revealed that the AMSTAR was slightly more time consuming in contrast to OQAQ; there may be several explanations for this. First, the AMSTAR has 11 items, longer than the 10 items in OQAQ; second, the AMSTAR was developed based on two instruments, including OQAQ itself, so items in the instruments may be overlapped, and it would take less time to complete scoring when filling replicated items; third, the sequence of applying tools may be another explanation, as we conducted the assessment in the order of first AMSTAR and then OQAQ, assessors need more time to look through a systematic review to facilitate it in the first round of assessment by using AMSTAR, while in the second round by applying OQAQ, such time would be saved.

The reliability analysis revealed that the Kappa was poor to fair in some items on AMSTAR. As kappa coefficient shows the proportion of agreement beyond that expected by chance alone, it is a popular measure for chance-corrected nominal scale agreement between two raters [5, 8]; however, if distribution of item responses is skewed or over concentrated on either the “yes” or the “no” category, the kappa coefficients will become unstable and invalid, and no longer suitable for measuring agreement between two raters, so new methods for calculating valid agreement coefficients are needed to explore in future study. Such items shown in our study included item 1 “was an ‘a priori' design provided,” item 6 “were the characteristics of the included studies provided,” item 7 “was the scientific quality of the included studies assessed and documented,” and item 8 “was the scientific quality of the included studies used appropriately in formulating conclusions”; however, the relatively low kappa in item 5 and item 9 cannot be explained by the limitation of kappa statistic.

AMSTAR proved to be feasible to apply in quality assessment of TCM systematic reviews; the main problems emerged were the absence of guidance for certain item response, such as item 5 “was a list of studies (included and excluded) provided,” to get “yes” score, four situations may be encountered: list of included studies provided, list of excluded studies provided, both lists of included and excluded studies provided in the same time, and the characteristics of the included studies presented; it is difficult to reach a final conclusion without more detailed directions regarding its use. Those items (4 and 7) with relatively low kappa values identified by Shea et al. [8], on the contrast, presented more precisely guidance, were easy to apply and easily reached consensus among raters.

As the assessment was undertaken by two assessors, one assessor was with expertise in clinical epidemiology and clinical research methods, and the other was a novice user to these quality assessment instruments, thus it could possibly result in underestimation of the reliability of AMSTAR. Another limitation in this study is lack of backward translation for the adapted tools, the translation into Chinese may produce a different measurement instrument with different properties. The current Chinese version should be translated back into English by a third party, and the back translations would be compared with the original tools to ensure the conceptual equivalence. However, the absence of back translation may offset somewhat in present study by a check of accuracy with a previous Chinese version of two instruments tools [10].

## 5. Conclusions

Although both instruments proved to be useful in this study, the performance of AMSTAR in terms of reliability and validity was better than OQAQ; the new tool is reliable, valid, and easy to use when applied to assess methodological quality of systematic reviews on TCM, although there is room for improvement on a few items.

## Figures and Tables

**Table 1:** Assessment of the interrater agreement for AMSTAR.

Item	Agreement (%, 95% CI)	Kappa (95% CI)
(1) Was an “a priori” design provided?	92.7 (80.1–98.5)	−0.03 (−0.10, 0.04)
(2) Was there duplicate study selection and data extraction?	85.4 (70.8–94.4)	0.70 (0.49, 0.91)
(3) Was a comprehensive literature search performed?	87.8 (73.8–95.9)	0.75 (0.55, 0.95)
(4) Was the status of publication (i.e., grey literature) used as an inclusion criterion?	95.1 (83.5–99.4)	0.72 (0.37, 1.00)
(5) Was a list of studies (included and excluded) provided?	48.8 (32.9–64.9)	0.16 (0.03, 0.30)
(6) Were the characteristics of the included studies provided?	78.1 (62.4–89.4)	0.40(0.08, 0.71)
(7) Was the scientific quality of the included studies assessed and documented?	92.7 (80.1–98.5)	0.36 (−0.20, 0.92)
(8) Was the scientific quality of the included studies used appropriately in formulating conclusions?	92.7 (80.1–98.5)	0.36 (−0.20, 0.92)
(9) Were the methods used to combine the findings of studies appropriate?	56.1 (39.8–71.5)	0.17 (0.01, 0.33)
(10) Was the likelihood of publication bias assessed?	97.6 (87.1–99.9)	0.95 (0.85, 1.00)
(11) Were potential conflicts of interest included?	100 (91.4-100)	1.00 (1.00, 1.00)
Total	84 (71.1–91.9)	0.50 (0.26, 0.73)
